# Is lung function in a race against time?

**DOI:** 10.1113/EP091490

**Published:** 2024-05-03

**Authors:** Brendan Cooper, Sanja Stanojevic

**Affiliations:** ^1^ Lung Function & Sleep Department, Queen Elizabeth Hospital University Hospitals Birmingham Birmingham UK; ^2^ Department of Community Health and Epidemiology Dalhousie University Halifax Nova Scotia Canada

It has been evident from the earliest studies of vital capacity that in addition to standing height, social class, occupation and factors that we now refer to as the social determinants of health have a profound influence on both lung size and function (Hegewald & Crapo, 2007; Kouri et al., 2021). Over nearly 200 hundred years, hundreds of research studies have highlighted that biological variability of lung function within and between populations persists even after adjusting for standing height. Several studies also demonstrated differences in vital capacity between racial groups, and attributed these to biological or innate factors (Braun et al., 2013; Wolff, 2006). This misconception perpetuated the belief that black individuals were inferior and justified slave practices for many decades (Braun, 2005; Wolff, 2006). Indeed, there are measurable differences in lung function between individuals of different geographical or ancestral backgrounds, but the causal mechanism are the result of a myriad exposures over the life course (Stocks et al., 2013). Critically, the vast majority of research studies that investigated differences in lung function between populations, or people of different racial and ethnic groups, did not account for socio‐economic differences (Braun et al., 2013). Even in high quality studies that account for socioeconomic differences, residual confounding due to multi‐generational epigenetic effects cannot be ruled out as possible contributors to the observed differences in lung function. A critical appraisal of historical literature and evidence to support the use of race/ethnicity‐specific reference equations for lung function led the American Thoracic Society to publish a statement calling for the practice to be ended (Bhakta et al., 2023).

Standing height is strongly correlated with lung size, and reference equations to predict how big the lungs ought to be for a given height have been essential tools to facilitate interpretation of lung function (Quanjer et al., 2012). The literature has, however, been systematically biased toward studies that only include people of European ancestry, which has skewed our understanding of lung growth and development. Even the largest study to date, the Global Lung Function initiative, is highly skewed toward people of European ancestry (80% of data) and does not represent the world's diverse population admixture (Bowerman et al., 2023; Quanjer et al., 2012). There is nothing about the European lung that makes it ideal or representative of what we should expect. This European‐centric perspective has led to the misconception that any differences observed between populations are biological or innate and reflect anatomical and physiological differences between individuals and populations. The terms ‘race’ and ‘ethnicity’ have been used as proxies for genetic, anatomical and physiological differences, and race‐specific or ethnic‐specific reference equations have been derived with the intent to facilitate more precise interpretation of measured values (Quanjer et al., 2012). The rationale proposed in more contemporary studies was that it is not lung size and function per se that differs between populations or racial/ethnic groups but that standing height is an imprecise proxy for chest size, and that observed differences in body proportions (i.e., Cormic index [sitting height: standing height ratio]) between populations explains the observed differences, and race and ethnicity can be used as proxies to account for those differences (Quanjer et al., 2012). Allen's Rule for all species, where for a given standing height, individuals from colder climates tend to have their lung function underestimated, while those from equatorial regions tend to have their lung function overestimated (Miller et al., 2022). It is unclear whether any of this evidence applies to today's multi‐cultural and geographically diverse population.

Further, the respiratory community has assumed that a group of individuals labelled as healthy (non‐smokers and not having persistent respiratory symptoms or any known lung disease) represent ideal or optimal lung function. Today, we better understand that many adverse exposures, including but not limited to tobacco smoke, influence the growth and development of the lungs. Early life factors such as nutrition, respiratory infections and air quality all are important influences on lung health. Further, many of these factors are deeply interwoven with geography and race and ethnicity. Reconciling racial bias in the interpretation of lung function will require a fundamental shift in our assumptions and understanding of the determinants of lung health and disease.

Race and ethnicity are social constructs that have different meanings and definitions across populations and time, and attributing an individual to these categories is fraught with bias (Vyas et al., 2020). Advancements in genomics have upturned historical theories of an ‘African race’; there are larger genetic differences identified within those from the African continent than between individuals from Africa and Europe (Borrell et al., 2021). Today with a much richer and broader understanding of the determinants of lung health we must recognize that we have perpetuated inequities and that social determinants of health are cumulative exposures across the life course (Bhakta et al., 2022). The historic white‐European centric evidence has led to an underestimation of lung disease for many people around the world. We must therefore question the utility of using an imprecise categorization of race and ethnicity, with poor correlation to genetic ancestry, as a justification for ‘improving’ the precision of lung function test interpretation (Marciniuk et al., 2023; Stanojevic et al., 2022).

## AUTHOR CONTRIBUTIONS

Both authors have read and approved the final version of this manuscript and agree to be accountable for all aspects of the work in ensuring that questions related to the accuracy or integrity of any part of the work are appropriately investigated and resolved. Both persons designated as authors qualify for authorship, and all those who qualify for authorship are listed.

## CONFLICT OF INTEREST

The authors declare no conflict of interest.

## FUNDING INFORMATION

No funding was received for this work.
